# Signatures of disease outcome severity in the intestinal fungal and bacterial microbiome of COVID-19 patients

**DOI:** 10.3389/fcimb.2024.1352202

**Published:** 2024-03-06

**Authors:** Fernando Rizzello, Elisa Viciani, Paolo Gionchetti, Eleonora Filippone, Veronica Imbesi, Laura Melotti, Nikolas Konstantine Dussias, Marco Salice, Barbara Santacroce, Antonella Padella, Alena Velichevskaya, Andrea Marcante, Andrea Castagnetti

**Affiliations:** ^1^ IBD Unit, IRCCS, Azienda Ospedaliero-Universitaria di Bologna, University of Bologna, Bologna, Italy; ^2^ Department of Medical and Surgical and Sciences, University of Bologna, Bologna, Italy; ^3^ Wellmicro srl, Bologna, Italy

**Keywords:** human microbiota, COVID-19, SARS-CoV-2, pathogenesis, mycobiota, microbiome, SCFA (short chain fatty acid)

## Abstract

**Background:**

COVID-19, whose causative pathogen is the Severe Acute Respiratory Syndrome Coronavirus-2 (SARS-CoV-2), was declared a pandemic in March 2020. The gastrointestinal tract is one of the targets of this virus, and mounting evidence suggests that gastrointestinal symptoms may contribute to disease severity. The gut–lung axis is involved in the immune response to SARS-CoV-2; therefore, we investigated whether COVID-19 patients’ bacterial and fungal gut microbiome composition was linked to disease clinical outcome.

**Methods:**

In May 2020, we collected stool samples and patient records from 24 hospitalized patients with laboratory-confirmed SARS-CoV-2 infection. Fungal and bacterial gut microbiome was characterized by amplicon sequencing on the MiSeq, Illumina’s integrated next generation sequencing instrument. A cohort of 201 age- and sex-matched healthy volunteers from the project PRJNA661289 was used as a control group for the bacterial gut microbiota analysis.

**Results:**

We observed that female COVID-19 patients had a lower gut bacterial microbiota richness than male patients, which was consistent with a different latency in hospital admittance time between the two groups. Both sexes in the COVID-19 patient study group displayed multiple positive associations with opportunistic bacterial pathogens such as *Enterococcus*, *Streptococcus*, and *Actinomyces*. Of note, the *Candida* genus dominated the gut mycobiota of COVID-19 patients, and adult patients showed a higher intestinal fungal diversity than elderly patients. We found that *Saccharomycetales unassigned* fungal genera were positively associated with bacterial short-chain fatty acid (SCFA) producers and negatively associated with the proinflammatory genus *Bilophila* in COVID-19 patients, and we observed that none of the patients who harbored it were admitted to the high-intensity unit.

**Conclusions:**

COVID-19 was associated with opportunistic bacterial pathogens, and *Candida* was the dominant fungal taxon in the intestine. Together, we found an association between commensal SCFA-producers and a fungal genus that was present in the intestines of patients who did not experience the most severe outcome of the disease. We believe that this taxon could have played a role in the disease outcome, and that further studies should be conducted to understand the role of fungi in gastrointestinal and health protection.

## Introduction

The COVID-19 pandemic, caused by the novel coronavirus, SARS-CoV-2, has had a profound global impact on public health and healthcare systems. Global research efforts have led to the rapid development and deployment of vaccines, which have been instrumental in reducing the incidence of severe disease and death (Atzrodt et al., 2020). However, the disease continues to evolve, with new variants of the virus emerging, posing additional challenges. Understanding the dynamics of infection is crucial for defining the factors influencing disease severity among infected individuals. COVID-19 presents a broad spectrum of clinical manifestations ranging from mild respiratory symptoms to severe pneumonia and acute respiratory distress syndrome (ARDS) (Gibson et al., 2020). Therapeutic strategies for COVID-19 are multifaceted and supportive. Hospitalized patients may require supplemental oxygen, and in severe cases, high-flow oxygen or mechanical ventilation in the ICU setting. Antiviral therapy and corticosteroids have been used in certain situations based on the severity of the illness (Wu et al., 2020). Age is a significant risk factor, with individuals aged 65 years and older having a higher risk. Comorbidities such as cardiovascular disease, diabetes, chronic respiratory diseases, obesity, and immunosuppression also significantly increase the risk. Additionally, socioeconomic factors and health inequalities have been shown to contribute to disease severity (Martono et al., 2023). The human gut microbiota (GM), a dynamic and heterogeneous ecosystem inhabited by microorganisms such as bacteria, fungi, archaea, and viruses, and the human host form a complex “super-organism” in which symbiotic relationships confer benefits to the host in many key aspects of life (Shreiner et al., 2015). GM has many significant functions in the human body, including supporting protection from pathogens by colonizing mucosal surfaces and creating different antimicrobial substances, enhancing the immune system, playing a vital role in digestion and metabolism, controlling epithelial cell proliferation and differentiation, modifying insulin resistance, affecting its secretion, influencing brain–gut communication, and thus affecting the mental and neurological functions of the host (Gomaa, 2020).

Variations and a reduction in the diversity of the gut microbiota can lead to a state of dysbiosis when stress conditions rapidly promote the expansion of commensal opportunistic pathogenic bacterial taxa (Weiss and Hennet, 2017). Emerging research suggests that gut microbiota may influence lung microbiota and have an impact on respiratory illnesses (Enaud et al., 2020). Alteration of the gut microbiota composition in patients with COVID-19 has been observed before ranging from reduced bacterial diversity to an increase in opportunistic pathogens, such as *Streptococcus*, *Rothia*, *Veillonella*, and *Actinomyces*, or *Collinsella aerofaciens*, *Collinsella tanakaei*, *Streptococcus infantis*, and *Morganella morganii*, and a lower relative abundance of beneficial commensal bacteria such as *Faecalibacterium prausnitzii*, *Eubacterium rectale*, and *Bifidobacterium* genus, with modifications in gut microbiome composition observed up to 30 days after recovery (Gu et al., 2020; Yeoh et al., 2021; Zuo et al., 2021). Beneficial short-chain fatty acid producers were also found to be higher in patients with less severe COVID-19 than in those with severe disease (Zuo et al., 2020). The study by Maeda et al. (2022) provided insight into the characteristics of the mycobiota and microbiota in COVID-19 severe patients, which displayed lower microbial diversity compared to the healthy group, and an increase in *Lactobacillus* and *Enterococcus*, positively correlated with the abundance of the fungal genus *Candida*, and a decrease in *Faecalibacterium* and *Bacteroides*. These studies will to investigate regarding how gut microbiota alterations can predict the clinical outcome of SARS-CoV-2 infection. However, further studies are necessary to confirm and bolster previous findings and to shed more light on the correlation between mycobiota and microbiota and the severity of COVID-19. Managing the gut microbiota of patients during and after COVID-19 to strengthen beneficial gut species, while fighting the increase in opportunistic pathogens, could serve as a novel approach to alleviate severe diseases. Therefore, in this study, we explored whether bacterial and fungal microbiome compositions were linked to disease outcome in patients hospitalized with COVID-19 during the first wave of the pandemic.

## Materials and methods

### Study population

The study included 24 Italian subjects who suffered from COVID-19 and were admitted to the SSD Malattie Infiammatorie Croniche Intestinali, IRCCS AOU di Bologna, Bologna, Italy, from April to May 2020. This study was conducted in accordance with the principles of the Declaration of Helsinki. The samples were coded and analyzed using an anonymized database. Upon admission to the hospital, the patient was informed about the possibility of participating in the clinical trial, “Biomarkers diagnostici e prognostici nelle infezioni da SARS-CoV-2,” and informed consent for study participation was obtained from each patient. The study was approved by the local IRB (Comitato Etico AVEC) with approval number n. 410.2020.Oss.AOUBo. The study population consisted of patients with a confirmed diagnosis of COVID-19 according to the World Health Organization definition (WHO, 2022) admitted to the SSD Malattie Infiammatorie Croniche Intestinali, IRCCS AOU Bologna, who met the following inclusion criteria: i) males or females ≥18 and ≤80 years of age; ii) confirmed diagnosis of SARS-CoV-2; diagnosis of acute COVID-19 infection was based on detection of SARS-CoV-2 RNA in respiratory specimens using real-time reverse transcription-polymerase chain reaction (RT-PCR); and iii) written informed consent to participate in the study. A healthy control dataset of gut microbiota samples that we selected from the available public peer-reviewed literature was used to compare bacterial COVID-19 patient gut microbiota samples, which met the following inclusion criteria for data analysis: i) age- and sex-matched adult subjects, ii) Italian origin, and iii) more than 7,000 sequencing reads each after quality-control filtering.

### Fecal sample collection and methodology

Fecal samples were collected from the enrolled patients to analyze the composition of their microbiome using biomolecular methods at the Wellmicro S.r.l. laboratory (Bologna). Fecal sampling was performed following spontaneous evacuation, as per the standard procedure in clinical practice, and samples were collected using the Copan eNat^®^ System sampling device (Copan). The samples were collected on the first day of hospitalization and stored in freezers at −80°C until analysis.

### DNA extraction and purification

Total microbial DNA was extracted from fecal samples using the DNeasy 96 PowerSoil Pro QIAcube HT Kit on a QIAcube HT instrument (QIAGEN, Hilden, Germany) according to the manufacturer’s instructions. A bead‐beating step with Lysing Matrix E (MP Biomedicals) was performed on a FastPrep24 bead-beater (MP Biomedicals, Irvine, CA) at 6.0 movements per second for 40 s, before total DNA extraction (Gaibani et al., 2021). The negative controls were PCR-grade water which underwent library preparation steps and Next Generation Sequencing (NGS), along with all the other samples. DNA was quantified using a Qubit™ 4 Fluorometer (Fisher Scientific) following the Illumina amplicon sequencing Sample Preparation Guide (https://emea.support.illumina.com/content/dam/illumina-support/documents/documentation/chemistry_documentation/16s/16s-metagenomic-library-prep-guide-15044223-b.pdf, accessed on the 8th of February, 2024).

### Determination of bacterial and fungal profiles by amplicon sequencing

Internal transcribed spacer 2 (ITS2) was amplified for fungal classification using the primer set ITS3: 5’-GCATCGATGAAGAACGCAGC-3’ and ITS4: 5’-TCCTCCGCTTATTGATATGC-3’ (White et al., 1990). The V3–V4 region of the 16S rRNA gene was amplified for bacterial classification by using the S‐D‐Bact‐0341‐b‐S‐17/S‐D‐Bact‐0785‐a‐A‐21 primer set (Klindworth et al., 2013). Indexed libraries were prepared by limited‐cycle PCR using Nextera technology (Illumina, San Diego, CA, USA) and further cleaned using VAHTS DNA Clean Beads (Vazyme, Red Maple Hi-tech Industry Park, Nanjing, PRC). Libraries were pooled at equimolar concentrations (4 nM), denatured, and diluted to 5 pM before loading onto a MiSeq Reagent Kit V2 (Illumina, San Diego, CA, USA). Sequencing on the MiSeq platform was performed using a 2 × 250 bp paired-end protocol according to the manufacturer’s instructions (Illumina, San Diego, CA, USA).

### Data processing and analysis

A dataset of 201 Italian age-matched healthy gut bacterial microbiota samples from the NU-AGE study (Tavella et al., 2021) was used to compare the COVID-19 patients bacterial gut microbiota features. The data were obtained from the European Nucleotide Archive (EMBL-EBI ENA) under the project ID PRJNA661289. For bacterial profiling, sequenced reads of COVID-19 patients from our study cohort and NU-AGE healthy control gut microbiota were analyzed using QIIME2 (Bolyen et al., 2019) (version 2020.6). The DADA2 (Hall and Beiko, 2018) (Divisive Amplicon Denoising Algorithm 2) plugin was used to remove noise and chimeras, and generate Amplicon Sequence Variants (ASVs). Quality filtering and clustering were performed using the VSEARCH software (Rognes et al., 2016). High‐quality reads were classified taxonomically using the SILVA reference database (Yilmaz et al., 2014), version 132. For ITS2 fungal profiling, paired-end sequenced reads were analyzed by combining PANDAseq2 (Masella et al., 2012) and QIIME2 version 2018.6. The Divisive Amplicon Denoising Algorithm 2 (DADA2) (Callahan et al., 2016a) plugin was used to remove noise and chimeras and generate Amplicon Sequence Variants (ASVs). Quality filtering, and clustering were performed using the VSEARCH software (Rognes et al., 2016). High‐quality reads were classified taxonomically using the UNITE reference database version 7.2 (UNITE Community (2017). Version 01.12.2017. UNITE Community. https://doi.org/10.15156/BIO/587481). For both fungal and bacterial data analysis, the data were imported into R (R Core Team, 2022) (version 4.2.2) in Rstudio (version 2022.07.2 Build 576) (RStudio Team, 2020), and downstream analysis was performed using the R packages *phyloseq* (McMurdie and Holmes, 2013; Callahan et al., 2016b), *rbiom*, *ggplot2* (Wickham, 2009), *tidyverse* (Paradis et al., 2004; Wickham et al., 2019)*, tidyr*, *ape* (Paradis et al., 2004), *ggpubr*, and *dplyr* (Wickham et al., 2023). Environmental microbial contaminants were excluded from the analysis by filtering out ASVs that were specifically present in the negative controls using the *decontam* R package at 5% stringency (Davis et al., 2018). We explored the sequencing depth of bacterial and fungal datasets using rarefaction curves. The rarefaction curve shows the number of new ASVs observed when new reads for a given sample are obtained. When the sequencing depth is sufficient, a plateau is observed, indicating that all the diversity present in a sample has already been described and that the ecosystem has been sequenced deeply enough. Based on the minimum sample sequence depth at which a plateau was reached on the rarefaction curve, it was possible to correct each sample for different sequencing depths and normalize the data by rarefaction without replacement. For bacterial data, the minimum sample sequence depth was 7,365 reads, whereas it was 4,574 reads for the fungal dataset. Of the 797 total fungal taxa, 279 were unassigned (35%). They were excluded from the downstream analysis, and the data were focused on the classified fraction of the taxa (n = 518, 65% of the fungal taxa). The differences in alpha diversity were evaluated, based on the data distribution of metrics, using ANOVA and Tukey’s HSD (honestly significant difference) tests for normally distributed data or Wilcoxon–Mann–Whitney with Holm–Bonferroni correction method (WMW with HB) for non-normally distributed data. To compare microbial composition between samples, beta diversity was measured by calculating the weighted or unweighted UniFrac distance matrix (Lozupone and Knight, 2005) on bacterial data and the Bray–Curtis distance matrix on fungal data. Principal coordinate analysis (PCoA) was applied to the distance matrices to generate bi-dimensional plots in R. The dispersion of PCoA clusters was compared using the *betadisper* function in the R *vegan* package (Oksanen et al., 2022). A permutational analysis of variance (PERMANOVA) test, calculated using the function *adonis2* in the *vegan* package (Oksanen et al., 2011), was performed to determine whether there was a significant separation between different sample groups. The linear discriminant analysis (LDA) effect size (LEfSe) algorithm (Segata et al., 2011), a tool hosted on the Galaxy web application at https://huttenhower.sph.harvard.edu/galaxy/, was used to identify bacterial or fungal taxa associated with COVID-19 patients. Differences in abundance were considered significant when the logarithmic LDA score was higher than 2.

### Statistical analysis

Permutational multivariate analysis of variance (PERMANOVA, 999 permutations) was used to test the differences among microbial beta diversity groups. Differentially abundant taxa were identified using the linear discriminant analysis (LDA) effect size (LEfSe) (Segata et al., 2011). Categorical variables were presented as counts and percentages, and continuous variables as median, minimum, and maximum values. For group comparisons, the Shapiro–Wilk’s test (Shapiro and Wilk, 1965) or the Kolmogorov–Smirnov (Massey, 1951) Test of Normality was used to test the data for normality assumptions. Fisher’s exact test (Fisher, 1922) was used to analyze categorical variables, the Mann–Whitney *U* test (Mann and Whitney, 1947) was used for non-normally distributed continuous data, and the *t*-test (Student, 1908)was performed on normally distributed continuous data.

## Results

### Lower gut bacterial microbiota richness in COVID-19 female patients

**Figure 1:**
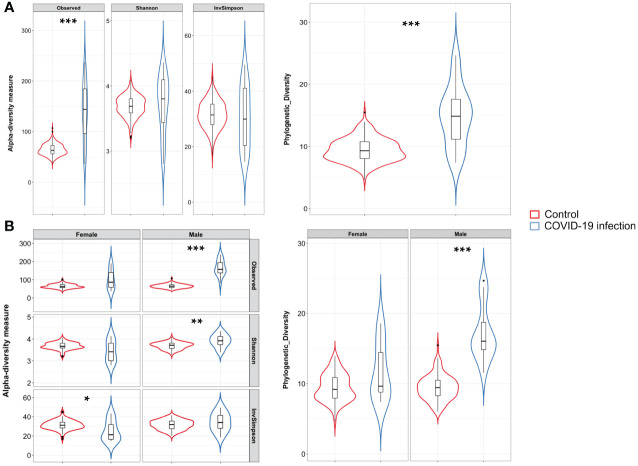
**(A)** Violin plots with box-and-whisker plot showing the comparison of alpha diversity measures between COVID-19 patients (n = 24, in blue) and controls (n = 24, in red). Observed Species p-value = 1.2 × 10^−8^; Phylogenetic Diversity Whole Tree p-value = 4.8 × 10^−8^, Wilcoxon–Mann–Whitney with Holm–Bonferroni (WMW with HB). Median, first, third quartile and outliers are shown. **(B)** Violin plots with box-and-whisker plot showing the comparison of alpha diversity measures between male COVID-19 patients (n = 14, in blue) and male controls (n = 100, in red), and female COVID-19 patients (n = 10, in blue) and female controls (n = 201, in red). Observed Species p-value = 2.3 × 10^−9^; Shannon–Wiener index p-value = 0.003; Phylogenetic Diversity Whole Tree p-value = 3.4 × 10^−9^; Inverse Simpson’s p-value = 0.039, WMW with HB. Median, first, third quartile and outliers are shown. Observed, Observed species index; Shannon, Shannon–Weiner index; InvSimpson, Inverse Simpson’s index. * = FDR-corrected p-value < 0.05; ** = FDR-corrected p-value < 0.01; *** = FDR-corrected p-value < 0.001.

A total of 24 Italian subjects diagnosed with COVID-19 and admitted to the SSD Malattie Infiammatorie Croniche Intestinali, IRCCS AOU di Bologna, Bologna, Italy, from April to May 2020 were included in the study. The clinical and demographic characteristics are presented in **Table 1**. Microbiome data from 201 Italian age-matched healthy subjects from the NU-AGE study (project ID PRJNA661289) (Tavella et al., 2021) were used to compare the COVID-19 patients bacterial gut microbiota features with a set of healthy subjects. First, we compared bacterial alpha-diversity between COVID-19 patients and healthy controls. We found that the Observed Species (ASVs) index values, indicating the richness of the ecosystem, in the healthy controls were lower than those in the COVID-19 cohort, as well as the Phylogenetic Diversity Whole Tree scores (**Figure 1A**). Moreover, by looking at the same alpha diversity indices while dividing the study groups by sex, we observed that the male patients in our dataset showed a significantly higher richness of species, evenness of distribution, and a higher phylogenetic diversity inside their gut microbiota compared to the healthy controls (**Figure 1A**), while the female patients showed a significantly lower evenness of distribution of the taxa with respect to the control group (see **Figure 1B**) and male patients (Inverse Simpson’s index p-value = 0.0008, ANOVA and Tukey’s HSD, data not shown). Based on the clinical data, we found that male patients waited for a significantly shorter time (**Table 1**) before going to the hospital; in fact, the median time to hospital admission was less than a week for male patients and more than two weeks for female COVID-19 patients (**Table 1**). Letting the SARS-CoV-2 infection go unchecked for a shorter time could have influenced the alpha diversity of the gut microbiota of male patients to a lesser extent than that of female patients (Clerbaux et al., 2022).

**Table 1:** Clinical characteristics of COVID-19 patients and NU-AGE healthy controls.

Variable	COVID-19 patients n = 24	Healthy controls n = 201	p-value
Age (median of years (min-max))	71 (26–95)	72 (65–79)	0.95
Sex (n (%) female (F), n (%) male (M))	10 (41.7), 14 (58.3)	101 (50.2), 100 (49.8)	0.52
Co-morbidity (n (%), F, M)	21 (87.5), 10, 11	N.A.	0.23
Oxygen therapy (n (%), F, M)	16 (66.7), 6, 10	N.A.	0.67
Discharged (n (%), F, M)	7 (29.2), 5, 2	N.A.	0.085
Transferred to low intensity unit (n (%), F, M)	11 (45.8), 3, 8	N.A.	0.24
Transferred to high intensity unit (n (%), F, M)	6 (25), 2, 4	N.A.	1
Elderly subjects (>65 years of age, n (%), F, M)	14 (58.3), 7, 7	201 (100), 101, 100	**<0.00001**
Elderly patients (>65 years of age, n (%), F/n female, M/n male)	14 (58.3), 7/10, 7/14	N.A.	0.4212
From onset to admittance (median of days (min–max) F, M)	15.5 (0–47), 6 (1–14) *	N.A.	**0.037**
From admittance to sampling (median of days (min–max) F, M)	6 (3–18), 5 (1–20) *	N.A.	0.81

Fisher’s exact test was used on categorical data, Mann–Whitney U two-tailed test for non-normally distributed continuous data, T-test two-tailed for normally distributed continuous data; data distribution was estimated with the Kolmogorov–Smirnov Test of Normality. N.A = not applied, significant values are highlighted in bold. * = eight out of 10 female patients and 13 out of 14 male patients gave information on this regard.

### COVID-19 patients’ gut microbiota confirms multiple associations with bacterial opportunistic pathogens

**Figure 2:**
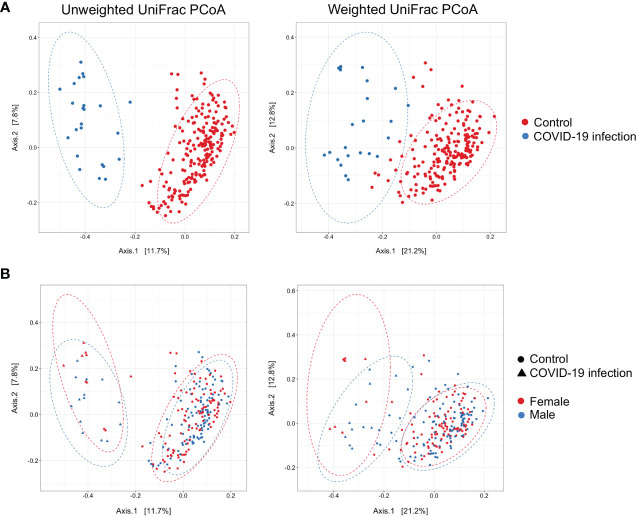
**(A)** Principal Coordinate Analysis (PCoA) on unweighted and weighted UniFrac distance metric calculated on COVID-19 patients (n = 24, blue dots) and healthy controls (n = 201, red dots). Each sample is represented by a dot. Axis 1 explained 11.7% and 21.2% of the variation observed, while Axis 2 explained 7.8% and 12.8% of the variation, in the left and right graph, respectively; PERMANOVA on weighted and unweighted UniFrac Pr(> F) = 0.001, beta-dispersion = 0.001. **(B)** Addition of the sex distinction layer to the same graphical display; PERMANOVA on weighted UniFrac Pr(> F) = 0.012, beta-dispersion = n.s.

**Figure 3:**
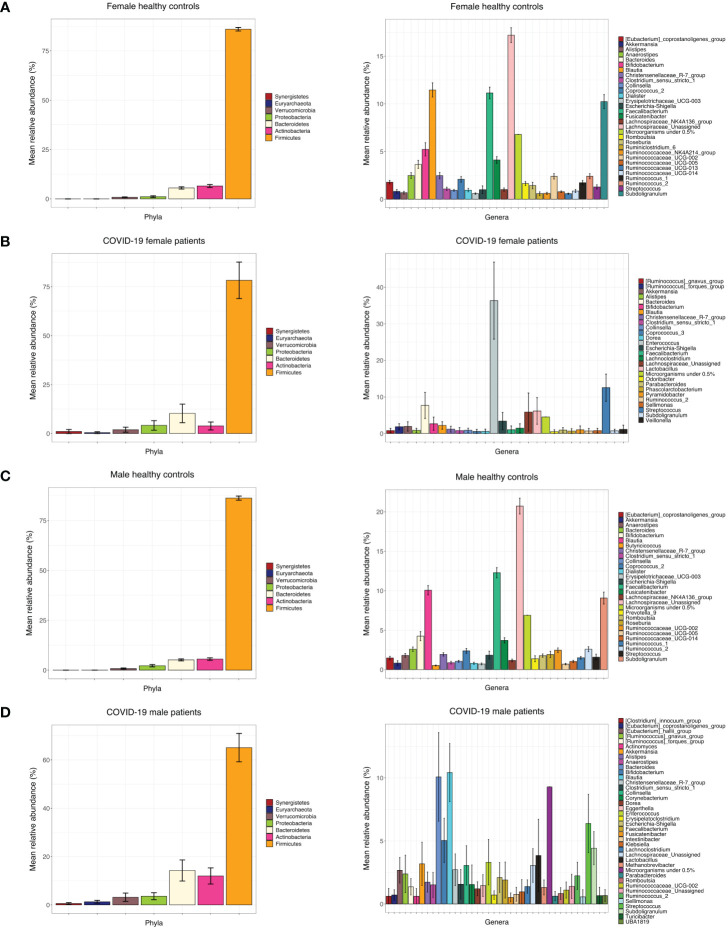
**(A)** Bar chart of mean RA% of bacterial phyla and genera in female control subjects. **(B)** Bar chart of mean RA% of bacterial phyla and genera in female COVID-19 patients. **(C)** Bar chart of mean RA% of bacterial genera and phyla in male control subjects. **(D)** Bar chart of mean RA% of bacterial genera and phyla in male COVID-19 subjects.

**Figure 4:**
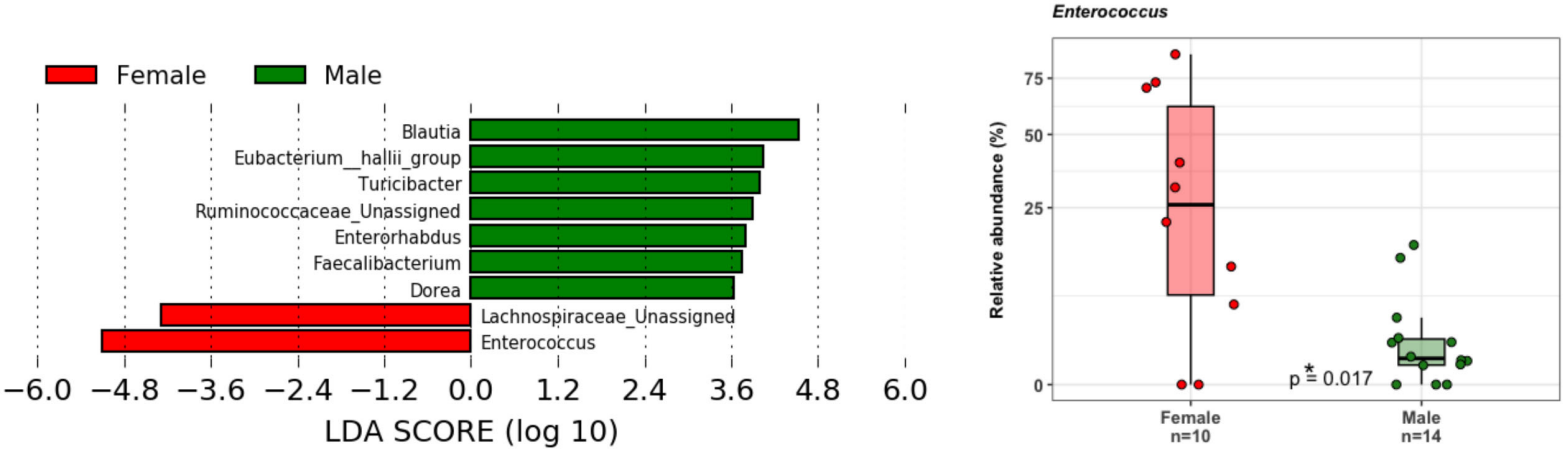
LDA LEfSe barplot displaying the different associations of bacterial genera between female (red) and male patients (green) (LDA score >2.0). Box-and-whisker plots with data points show the relative abundances of *Enterococcus* genus in the two groups. Median, first, third quartile are shown. Mann–Whitney *U* Test result of the group comparison is shown.

**Figure 5:**
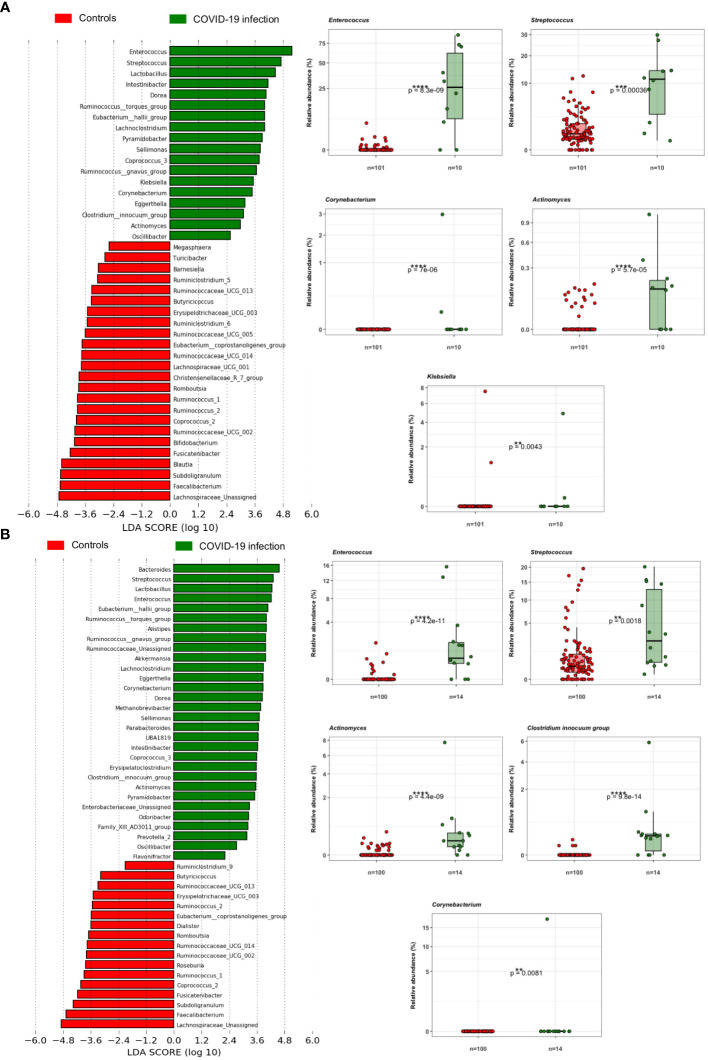
**(A)** LDA LEfSe barplot showing the different associations of gut bacteria between female patients and female controls (LDA score >2.0). Box-and-whisker plots with data points show the relative abundances of each potential opportunistic pathogen positively associated with the female patients. Mann–Whitney *U* Test results of the group comparisons are shown. **(B)** LDA LEfSe barplot showing the different associations of bacteria between male patients and male controls (LDA score >2.0). Box-and-whisker plots with data points show the relative abundances of each potential opportunistic pathogen positively associated with the male patients. Median, first, and third quartiles are shown in the box-and-whisker plots. Mann–Whitney *U* Test results of the group comparisons are shown.

Principal coordinate analysis (PCoA) of unweighted and weighted UniFrac distances showed distinct clustering of healthy controls and COVID-19 patients with samples combined with a different dispersion of the data (**Figure 2A**), highlighting the fact that the bacterial gut microbiota composition of the two sets of samples presented dissimilarities. Based on previous observations, we reported on the alpha diversity characteristics of the dataset and verified whether the data composition of the two groups had a different clusterization based on sex (**Figure 2B**). The unweighted UniFrac analysis did not find any separation between the two groups, while the weighted UniFrac, which also considers the relative abundance of the taxa that belong to every sample and, unlike the unweighted UniFrac, is more sensitive to changes in the most abundant taxa than in the rare taxa, there was a significant separation in the multivariate space between female and male patients (**Figure 2B**). Among the bacterial phyla, Firmicutes, Bacteroidetes, Actinobacteria, Proteobacteria, and Verrucomicrobia were the dominant taxa in both controls and COVID-19 patients (**Figure 3**). In female subjects, an increase in the Bacteroidetes mean relative abundance (mean RA%) in the COVID-19 patients (10.3%) and Proteobacteria (4.1%) was accompanied by a slight reduction in Firmicutes (78.3%), compared to the female controls (**Figures 3A, B**). In male subjects, Firmicutes mean RA% was reduced (65.1%) in favor of an expansion of Bacteroidetes (14.3%), Actinobacteria (12%), and Verrucomicrobia (3.2%) (**Figures 3C, D**). Female COVID-19 patients’ mean RA% composition at the genus level was *Enterococcus* (36.3%), *Streptococcus* (12.5%), *Bacteroides* (7.7%), *Lactobacillus* (6.2%), *Lachnospiraceae unassigned* (5.9%), and *Escherichia–Shigella* (3.4%), whereas female controls had a predominance of *Lachnospiraceae unassigned* (17.2%), *Blautia* (11.5%), *Faecalibacterium* (11.1%), *Subdoligranulum* (10.2%), *Bifidobacterium* (5.2%), *Fusicatenibacter* (4.1%), and *Bacteroides* (3.6%). Male COVID-19 patients mean RA% of gut bacteria at the genus level was characterized by *Blautia* (10.4%), *Bacteroides* (10.1%)*, Streptococcus* (6.4%), *Bifidobacterium* (5.04%), *Subdoligranulum* (4.4%), *Lactobacillus* (3.8%), *Enterococcus* (3.3%), *Akkermansia* (3.2%), *Collinsella* (3.1%), and *Lachnospiraceae unassigned* (3.1%), whereas the average gut microbiota of male healthy controls was characterized by *Lachnospiraceae unassigned* (20.7%), *Faecalibacterium* (12.3%), *Blautia* (10.1%), *Subdoligranulum* (9.1%), *Bifidobacterium* (4.2%), and *Fusicatenibacter* (3.7%) (**Figures 3C, D**). We further assessed the bacterial signatures associated with SARS-CoV-2 infection by LDA LEfSe analysis. With an LDA cutoff score of 2.0, we identified nine bacterial genera that were differentially associated with the two conditions (**Figure 4**). We found that the bacterial gut microbiota of female patients was positively associated and largely dominated by *Enterococcus*, whereas that of male patients was associated with other common bacterial gut commensals. We then analyzed the gut microbiota of COVID-19 patients in comparison with that of healthy controls. We identified 47 and 42 taxa enriched in male patients versus male controls and female patients versus female controls, respectively (**Figures 5A, B**). In particular, the analysis showed a positive association between female patients and potential opportunistic bacterial genera such as *Enterococcus*, *Streptococcus*, and *Actinomyces*, which were instead negatively associated with the gut microbiota of healthy females. We also found a positive association between COVID-19 gut microbiota and *Klebsiella* (Jiang et al., 2022) and *Corynebacterium*; however, in these cases, the results were clearly driven by two outliers in both study groups despite the significance of the statistical test (**Figure 5A**). Similar potential opportunistic genera were found in the male subject data set, such as *Enterococcus*, *Streptococcus*, *Clostridium innocuum* (Chia et al., 2017), and *Actinomyces*. We also detected the *Corynebacterium* genus to be positively associated with male COVID-19 patients; however, as shown in **Figure 5B**, in this case, the result was determined by an outlier in the male patient group despite the significance of the statistical test. These results highlight the fact that female and male patients had positive associations with many opportunistic pathogens compared to healthy controls (**Figure 5**).

### COVID-19 adult patients show a higher fungal diversity compared to elderly patients

**Figure 6:**
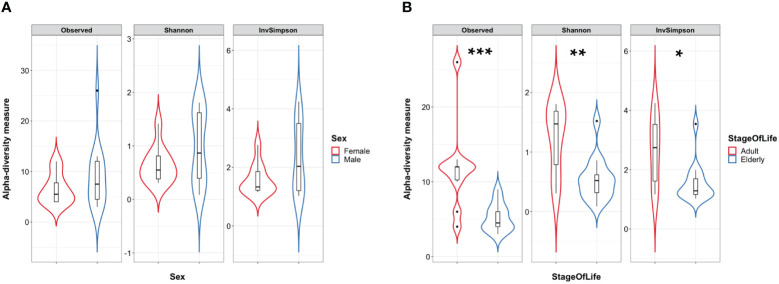
**(A)** Violin plots with box-and-whisker plot showing the comparison of fungal alpha diversity measures between COVID-19 female patients (n = 10, in red) and male patients (n = 14, in blue). **(B)** Violin plots with box-and-whisker plot showing the comparison of fungal alpha diversity measures between COVID-19 adult patients (n = 10, in red) and elderly patients (n = 14, in blue). Observed p-value = 0.001; Shannon p-value = 0.006; InvSimpson p-value = 0.013. Median, first, third quartile and outliers are shown. Observed, Observed species index; Shannon, Shannon–Wiener index; InvSimpson, Inverse Simpson’s index. * = FDR-corrected p-value < 0.05; ** = FDR-corrected p-value < 0.01; *** = FDR-corrected p-value < 0.001.

At the time of writing, we could not find a public database of gut mycobiome data of healthy subjects that met our inclusion criteria: i) age and sex-matched adult subjects, ii) Italian origin, and iii) more than 4,000 sequencing reads each after QC filtering; thus, we will describe the fungal observations we collected based on the COVID-19 patients database alone. To analyze the COVID-19 patient mycobiome, we first explored fungal alpha diversity between female and male patients to assess whether sex-based differences were present in the fungal microbiome data, but we could not find differences in fungal alpha diversity values based on sex (**Figure 6A**). This allowed us to further stratify the fungal dataset. We could not find differences between COVID-19 patients who did or did not undergo oxygen therapy. No significant differences between the fungal alpha diversity index values were found when comparing samples from patients who had been discharged, admitted to the low-intensity unit, or admitted to the high-intensity unit (**Supplementary Figures 1A, B**). Conversely, when we compared the elderly patients to the adult COVID-19 patients, we observed that the elderly patients in our dataset showed a lower species richness and evenness of distribution in their fungal gut microbiota than the adult patients (see **Figure 6B**). PCoA based on the Bray–Curtis distance matrix between the above-described subgroups (sex, stage of life, oxygen therapy, or outcome) of COVID-19 patients did not show any separation into distinct clusters, suggesting a similar trend between the mycobiome profiles of the subgroups analyzed (**Supplementary Figure 2**).

### COVID-19 patients with *Saccharomycetales unassigned* fungal genus are positively associated with bacterial SCFA-producers

**Figure 7:**
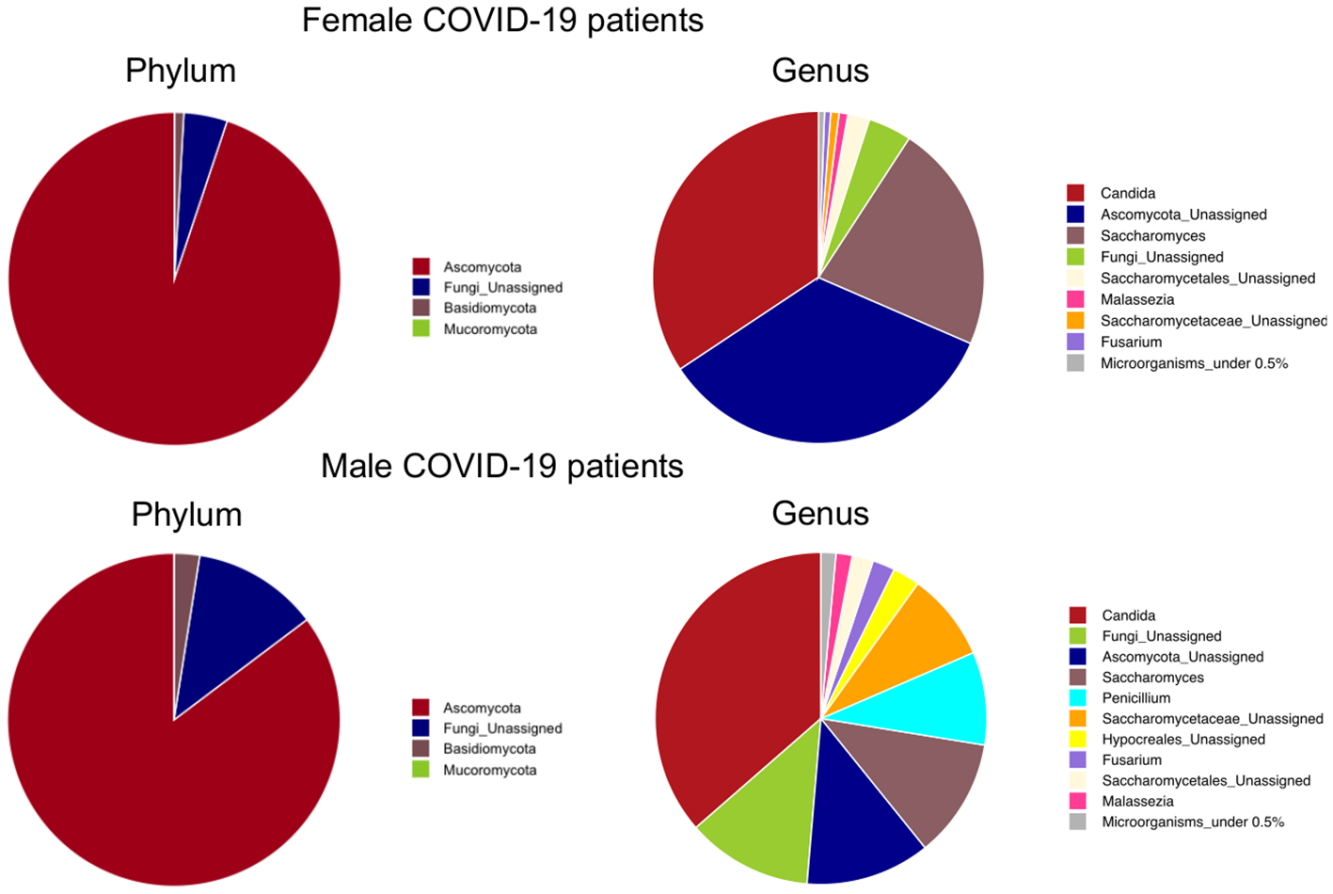
Phylum-level and genus-level mean relative abundance percentage pies of female and male COVID-19 patients’ fungal taxa.

**Figure 8:**
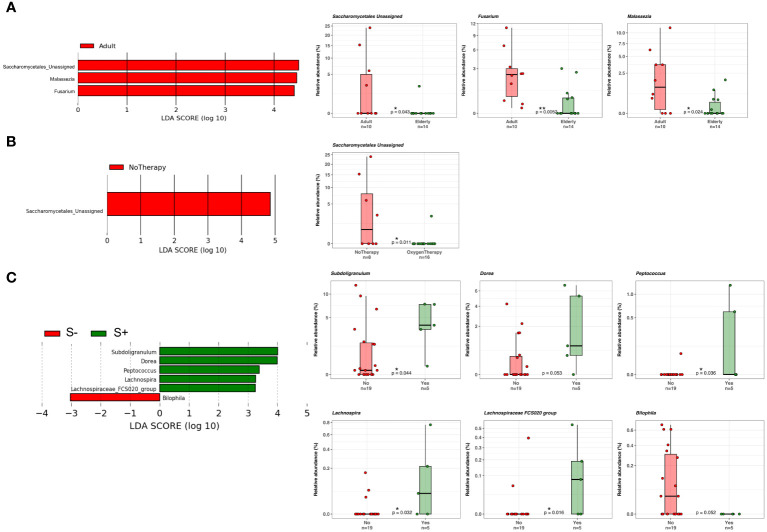
**(A)** LDA LEfSe barplot showing the different associations of fungi between adults (n = 10) and elderly patients (n = 14) (LDA score >2.0). **(B)** LDA LEfSe barplot showing the different associations of fungi between patients who did (n = 8) or did not (n = 16) undergo oxygen therapy (LDA score >2.0). **(C)** LDA LEfSe barplot showing the different associations of fungi between S+ (*Saccharomycetales unassigned* positive, n = 5) or S− patients (n = 19) (LDA score >2.0). On the right, from the top to the bottom of the figure, boxplots with data points show the relative abundances of each taxon displayed on the LDA LEfSe graphs on the left. Median, first, and third quartiles are shown in the box-and-whisker plots. Mann–Whitney *U* Test results of the group comparisons are shown.

At the phylum level, as shown in **Figure 7**, Ascomicota dominated the fungal ecosystems of female (94.9%) and male (85.3%) COVID-19 patients, followed by the Fungi unassigned (4.2% and 12.2%, respectively) and Basidiomycota (0.9% and 2.5%, respectively). Among the fungal genera, *Candida* dominated both the female (34.3%) and male (36.4%) gut mycobiota, followed by the *Ascomycota unassigned* (34.2%) and *Saccharomyces* (22.3%), which were the dominant fungal genera in the female gut mycobiota, and the *Fungi unassigned* (12.2%), *Ascomycota unassigned* (12.1%), *Saccharomyces* (11.6%) and *Penicillium* (9.1%) in the male gut mycobiota (**Figure 7**). LEfSe (LDA ≥2.0) identified fungal signatures associated with the stage of life (adulthood versus seniority) of COVID-19 patients, where adults were positively associated with fungal genera *Saccharomycetales unassigned*, *Fusarium*, and *Malassezia* (**Figure 8A**). By comparing the fungal gut microbiota of patients who underwent oxygen therapy against those who were not subjected to this treatment, LEfSe (LDA ≥2.0) found that *Saccharomycetales unassigned* was also associated with the group of patients who did not undergo oxygen therapy and was present in four out of eight patients (50%) (**Figure 8B**). Since *Saccharomycetales* spp. are known have a positive association with bacterial short-chain fatty acid (SCFA) producers *Clostridium sensu stricto* 1, *Faecalitalea*, and *Megamonas* (Shuai et al., 2022), we reassessed the data on the basis of the presence or absence of this fungal taxon in COVID-19 patients. A total of five out of 24 patients, two female and three male patients, four adults (26, 46, 55, and 62 years of age) and one elderly patient (86 years of age), on average, not older than the rest of the patients, had *Saccharomycetales unassigned* fungal taxon (S+) and 19 did not (S−). Based on the bacterial associations that the S+ patients had, we found that they were positively associated with *Peptococcus*, a genus of intestinal bacterial commensals, some of which can turn into opportunistic pathogens such as *Peptococcus magnus* (Wu et al., 2017), but also with *Subdoligranulum*, *Lachnospira*, *Dorea*, and *Lachnospiraceae FCS020* group, which are considered bacterial SCFA-producers (Lee et al., 2021; Nogal et al., 2021; Nishiwaki et al., 2022) (**Figure 8C**), whereas the S− patients were associated with *Bilophila*, a bacterial commensal genus whose abnormal increase has been associated with proinflammatory states, Inflammatory Bowel Disease (IBD), or colon cancer (without causal correlation) (Garrett and Onderdonk, 2014; Dekker Nitert, 2019). Moreover, three out of five (60%) S+ patients, while four out of 19 (21%) S+ patients were discharged, two out of five (40%) S+ patients, and nine out of 19 (47%) S− patients were admitted to the low-intensity unit, but none of the S+ patients were admitted to the high-intensity unit (0%), while six out of 19 (32%) S− subjects were transferred to this unit. In addition, as stated before, four out of five S+ subjects did not undergo oxygen therapy (80%), while only four out of 19 (21%) of the S− patients were not subjected to this treatment.

## Discussion

This study investigated the intestinal bacterial and fungal components of COVID-19 patients based on fecal samples collected upon admission to the hospital premises with the aim of exploring possible prognostic fungal and bacterial biomarkers of disease outcome. Previous studies have highlighted the importance of a healthy gut microbiome as a protective element against COVID-19 onset and identified a positive correlation between intestinal commensal depletion and COVID-19 severity during hospitalization (Zuo et al., 2020; De and Dutta, 2022). Unexpectedly, we found that the richness of the ecosystem in the healthy controls was lower than that in the COVID-19 cohort, as well as the Phylogenetic Diversity (**Figure 1A**). As indicated in **Table 1**, the proportion of elderly patients was significantly lower in the COVID-19 group than in the controls; because age influences the richness of the gut microbiota (Ghosh et al., 2022) this result could be explained by this characteristic of the two datasets. Indeed, we also observed a decrease in the evenness of the biodiversity intrinsic to the gut microbiota of female patients when compared to female healthy controls, who appeared to have been infected for a longer time before hospital admission, confirming a more severe disruption of the gut microbiota composition in female patients in our dataset. Looking at the bacterial biomarkers that we found in female COVID-19 patients, we observed a positive association with *Enterococcus*, *Streptococcus*, and *Actinomyces* and a negative association with many intestinal beneficial commensals such as *Faecalibacterium* and *Bifidobacterium*, signatures that have been previously found to be linked with COVID-19 severe cases (Gu et al., 2020; Zuo et al., 2020, 2021; Yeoh et al., 2021; Maeda et al., 2022). Moreover, these patients were positively associated with *Enterococcus*, a COVID-19 bacterial signature, when compared to male patients, demonstrating the presence of an even higher relative abundance of this opportunistic pathogenic genus in their intestinal ecosystem. Regarding the fungi in the human gut little is known about their role in COVID-19 infection since most previous studies have focused on the more abundant bacterial component. The gut mycobiome is a component of the gut microbiome in humans; it represents a small portion of the total intestinal microorganisms but plays a critical role in the regulation of host homoeostasis, pathophysiological and physiological processes, and in the organization of the gut bacterial microbiome co-residents (Zhang et al., 2022). *Candida*, one of the most common fungal gut commensal genera (Nash et al., 2017), was found in our dataset as the dominant fungal genus in the intestines of female and male COVID-19 patients. In our dataset, the median patient age was 71 years old. This observation is consistent with the fact that a *Candida-*dominated gut ecosystem is usually found in the elderly population and is associated with human ailments linked to a compromised intestinal epithelial barrier (Lai et al., 2023). In parallel, we discovered that *Malassezia*, *Saccharomycetales unassigned*, and *Fusarium* were biomarkers of the gut mycobiota of adult patients. *Malassezia* is normally found like *Saccharomyces* in the healthy gut (Nash et al., 2017). The fungal genus *Fusarium* is a gut commensal, but it can harbor opportunistic pathogenic fungi, such as *Fusarium proliferatum*, a causative agent of human respiratory disorders (Lv et al., 2021). In the case of systemic inflammation, as it can occur during COVID-19 progression, the gut barrier can be disrupted, and the enrichment of fungal commensals, which can become opportunistic pathogens, such as *Candida* and *Fusarium*, increases the risk of fungal coinfections in other body districts, as described by Zhang et al. in SARS-CoV2 severely affected patients in 2020 (Zhang et al., 2020; Kusakabe et al., 2023). *Saccharomycetales unassigned* (genus level) has been found to be associated with short-chain fatty acid (SCFA)-producing bacteria in the human intestine (Shuai et al., 2022). SCFAs are gut bacteria-derived metabolites used by the human host as energy substrates for colonocytes and peripheral tissues. In our study, for the first time, we confirmed that this fungal taxon has a positive association with bacterial SCFA-producers during SARS-CoV-2 infection, such as *Subdoligranulum*, *Lachnospira*, *Dorea*, and *Lachnospiraceae FCS020* group, reported bacterial SCFA-producers (Lee et al., 2021; Nogal et al., 2021; Nishiwaki et al., 2022), in *Saccharomycetales unassigned*-positive (S+) COVID-19 patients. At the same time, we also showed that the S+ patients were negatively associated with *Bilophila*, whose increase has been associated in literature with Inflammatory Bowel Disease (IBD) or colon cancer (without causal correlation) (Garrett and Onderdonk, 2014; Dekker Nitert, 2019). Unfortunately, the sample size of our study did not allow us to apply machine learning-based prediction models to our data for COVID-19 outcome prediction, because of the low number of *Saccharomycetales unassigned*-positive (S+) COVID-19 patients that we reported. Our study would benefit from the addition of more sample data from other similar studies to confirm and strengthen our findings; therefore, our investigation can be defined as an explorative study.

Another limitation of the present study is the absence of a suitable healthy control group for comparison of fungal microbiota composition. Despite the existence of available fungal amplicon sequencing studies on healthy people (Nash et al., 2017), we could not find fungal data of Italian origin from healthy gut samples, and age and sex matched with our dataset. These limitations reflect the reality of performing studies during the major pandemic wave of the first half of 2020 when hospital wards were overwhelmed with COVID-19 patients, financial resources were redirected to improve and sustain the emergency units, and research funds were momentarily restricted. In addition, very few contemporary healthy subjects were available for analysis at our center. Further larger prospective studies should be performed to compare the microbiome and mycobiome of COVID-19 patients against contemporary healthy subjects, since new data could help corroborate our results and previous findings. In addition, the enrichment of fungal taxonomy databases could help to understand intestinal fungal ecology more deeply by improving the classification of taxa in mycobiome studies, thus providing scientists with the potential to unravel the characteristics of the intestinal gut mycobiome to a greater extent. A large number of healthy bacterial gut microbiota samples (201) were used to compare a cohort of 24 COVID-19-positive patient bacterial gut microbiota samples, an approach that may appear unbalanced. However, evaluation of the sample size in microbiome studies remains a critical step (Ferdous et al., 2022). Microbiome data have high dimensionality and high variability in counts between samples and are very sparse. Notably, the healthy gut microbiota changes widely across healthy people, causing high interindividual variability (McBurney et al., 2019); hence, it is necessary to consider as many suitable healthy gut microbiota sample data as possible. Another factor that should be taken into account is that the results from our study come from samples originating from a specific geographic region; changes in compositions observed here may not necessarily be reflected in COVID-19 patients from other countries. Moreover, since the median age of the COVID-19 patients was 71 years, it would be important to assess whether an association between bacterial SCFA-producers and fungal taxa, as the one described in our study, also exists in younger COVID-19 patients, to provide more insights into the possibility of using SCFA or SCFA-producer stimulations in therapeutic approaches.

This survey of gut microbiota alterations in association with COVID-19 clinical outcomes showed that gut bacteria and fungi are likely involved in the well-being of patients with COVID-19 and that patients who did not experience severe outcomes had a higher abundance of intestinal bacterial SCFA-producers. To our knowledge, this is the first study to confirm the association of *Saccharomycetales unassigned* to SCFA-producing beneficial bacteria during COVID-19, supporting the hypothesis that fungal and bacterial signatures together may be important in the prediction of disease severity in COVID-19. This observation paves the way for the concept that prebiotic administration to sustain SCFA-producing bacterial populations could be a possible approach to decreasing COVID-19 sequelae (Ngo and Gewirtz, 2021). Thus, these findings underscore the urgent need to confirm the specific roles of gut microorganisms in human immune and systemic responses to viral infection and to understand what foods and supplements can support clinical treatments and help counteract the complications of infectious diseases. Further studies with larger cohorts of patients are necessary to better understand these complex relationships.

## Data availability statement

The datasets presented in this study can be found in online repositories. The names of the repository/repositories and accession number(s) can be found here: https://www.ncbi.nlm.nih.gov/bioproject/PRJNA1048318.

## Ethics statement

The studies involving humans were approved by the Hospital Ethics Committee “Area Vasta Emilia-Centro,” Comitato Etico Area Vasta Emilia Centro (AVEC). The studies were conducted in accordance with the local legislation and institutional requirements. All the participants provided written informed consent to participate in study.

## Author contributions

FR: Conceptualization, Writing – original draft, Writing – review & editing, Project administration, Resources, Supervision. EV: Data curation, Formal Analysis, Software, Visualization, Writing – original draft, Writing – review & editing, Conceptualization, Methodology. PG: Conceptualization, Project administration, Supervision, Writing – review & editing, Investigation, Methodology, Resources. EF: Writing – review & editing, Methodology. VI: Conceptualization, Project administration, Supervision, Writing – review & editing, Investigation, Methodology. LM: Writing – review & editing. ND: Conceptualization, Project administration, Supervision, Writing – review & editing, Investigation. MS: Writing – review & editing. BS: Methodology, Writing – review & editing. AP: Methodology, Writing – review & editing. AV: Methodology, Writing – review & editing. AM: Methodology, Writing – review & editing. AC: Conceptualization, Project administration, Resources, Supervision, Writing – review & editing, Investigation, Methodology.

## Supplementary material

Supplementary Figure 1 **(A)** Violin plots with box-and-whisker plot showing the comparison of fungal alpha diversity measures between COVID-19 patients who underwent oxygen therapy (n = 16, in blue) and who did not receive oxygen therapy (n = 8, in red). **(B)** Violin plots with box-and-whisker plot showing the comparison of fungal alpha diversity measures between COVID-19 patients who were discharged, admitted to low intensity unit or to high intensity unit. Median, first, third quartile and outliers are shown. Observed = Observed species index; Shannon = Shannon-Wiener index; InvSimpson = Inverse Simpson’s index.
