# Supplementary figures and images for: Signatures of disease outcome severity in the intestinal fungal and bacterial microbiome of COVID-19 patients
Source: Front Cell Infect Microbiol. 2024 Mar 6;14:1352202. doi: 10.3389/fcimb.2024.1352202 (PMC10952111; doi:10.3389/fcimb.2024.1352202)

A

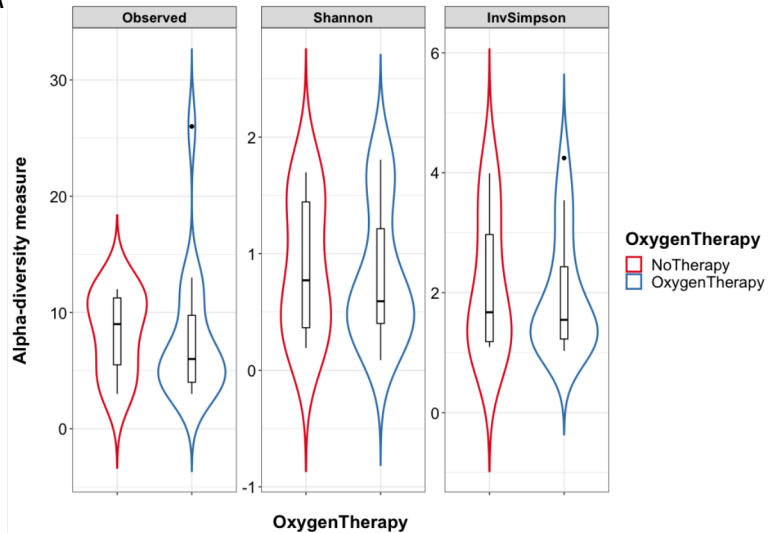

B

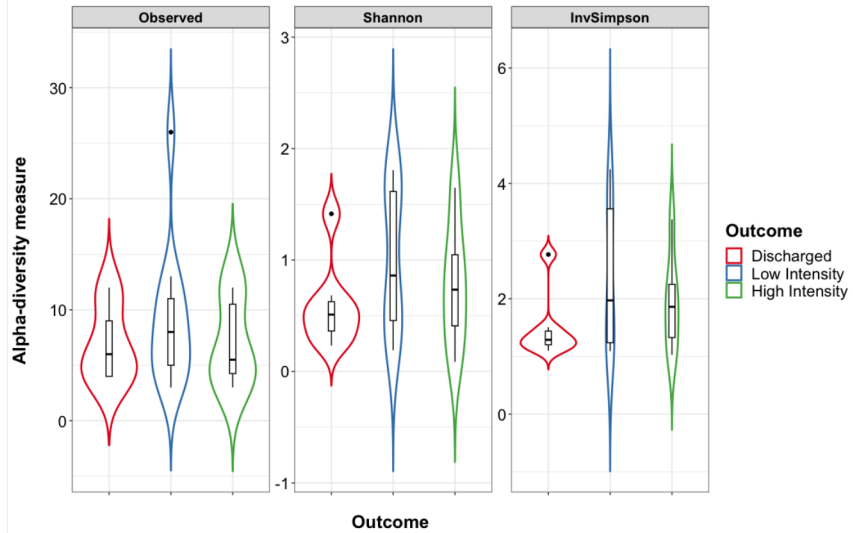

Supplement: Supplementary Figure 1 — (A) Violin plots with box-and-whisker plot showing the comparison of fungal alpha diversity measures between COVID-19 patients who underwent oxygen therapy (n = 16, in blue) and who did not receive oxygen therapy (n = 8, in red). (B) Violin plots with box-and-whisker plot showing the comparison of fungal alpha diversity measures between COVID-19 patients who were discharged, admitted to low intensity unit or to high intensity unit. Median, first, third quartile and outliers are shown. Observed = Observed species index; Shannon = Shannon-Wiener index; InvSimpson = Inverse Simpson’s index. [file Image_1.pdf]
